# Adherence to the “Mediterranean Diet” in Spain and Its Relationship with Cardiovascular Risk (DIMERICA Study)

**DOI:** 10.3390/nu8110680

**Published:** 2016-10-28

**Authors:** José Abellán Alemán, María Pilar Zafrilla Rentero, Silvia Montoro-García, Juana Mulero, Alfonso Pérez Garrido, Mariano Leal, Lucía Guerrero, Elena Ramos, Luis Miguel Ruilope

**Affiliations:** 1 Departamento de Nutrición y Cátedra de Riesgo Cardiovascular, Facultad de Ciencias de la Salud, UCAM Universidad Católica San Antonio de Murcia, Campus de los Jerónimos, s/n, Guadalupe 30107, Murcia, Spain; jabellan@ucam.edu (J.A.A.); mpzafrilla@ucam.edu (M.P.Z.R.); jmulero@ucam.edu (J.M.); aperez@ucam.edu (A.P.G.); arboleja@yahoo.es (M.L.); 2 Grupo EHRICA (Enfermería de Hipertensión y Riesgo Cardiovascular) Hospital 12 de Octubre, Madrid 28041, Spain; lucia.guerrero@ehrica.org (L.G.); elena-rq@telefonica.net (E.R.); 3 Instituto de Investigación y Unidad de Hipertensión, Hospital 12 de Octubre, Madrid 28041, Spain; ruilope@ad-hocbox.com

**Keywords:** adherence, cardiovascular risk factors, Mediterranean diet, Spain, score

## Abstract

Background: Nutritional studies focus on traditional cultural models and lifestyles in different countries. The aim of this study was to examine the adherence to the Mediterranean diet, life habits, and risk factors associated with cardiovascular diseases among people living in different geographical regions in Spain. Methods: A descriptive cross-sectional study was conducted in each region. The sampling scheme consisted of a random three-stage stratified sampling program according to geographic region, age, and gender. A total of 1732 subjects were asked to complete a questionnaire designed to assess their nutrient intake, dietary habits, and exercise. A diet score that assesses the adherence of participants to the Mediterranean diet (range 0–10) was also applied. Results: Southeastern Spain had the lowest score for adherence to the Mediterranean diet because of the low consumption of fish and plant products. A lower adherence score to the Mediterranean diet was strongly associated with the prevalence of hypertension (*p* = 0.018). Conclusions: A low level of adherence to the Mediterranean diet is accompanied by a high prevalence of hypertension and, therefore, a raised cardiovascular risk in the country. The adherence score could help identify individuals at greater cardiovascular risk.

## 1. Introduction

Cardiovascular disease (CVD) in Europe has traditionally been related to risk factors; however a significant body of data has revealed another layer of complexity in the problem, pointing to the need for a cardio healthy diet and regular exercise for increased protection against cardiovascular diseases, while genetics also play a role [1,2]. In this respect, a long history of epidemiological studies has suggested that the Mediterranean diet (MD) is associated with protection against cardiovascular heart disease, obesity, and cancer, as well as with reduced prevalence of Parkinson’s and Alzheimer’s diseases [3,4,5]. Moreover, several meta-analyses of cohort and follow-up studies further confirm such associations between the Mediterranean diet, health status, and fatal/nonfatal cardiovascular disease [6,7,8].

**Figure 1:**
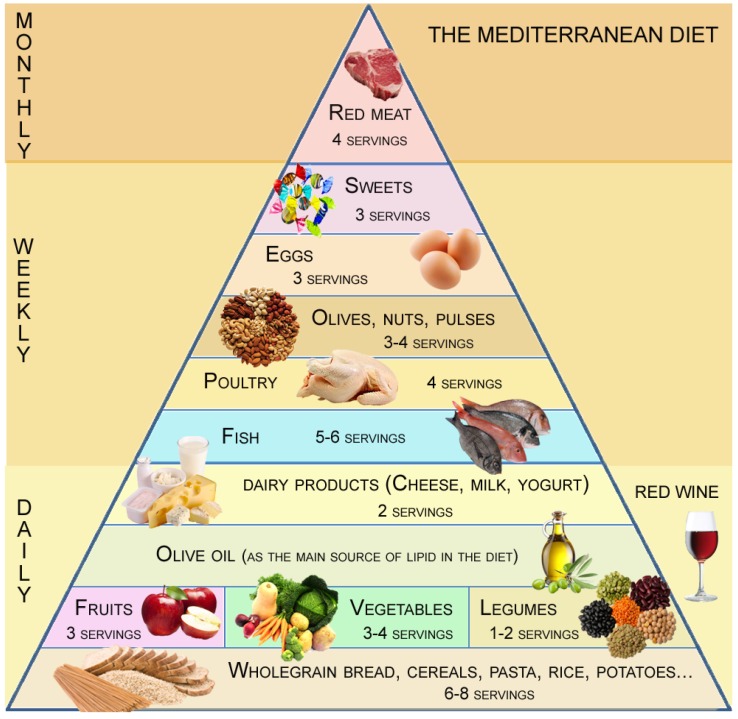
The Mediterranean diet food pyramid with servings.

Accordingly, the MD has been proposed as the best “healthy-heart eating model” and is recommended by clinicians. The MD is defined as a daily source of plant foods (fruits, vegetables, legumes, cereals, and seeds), moderate amounts of dairy and saturated fat products combined with a high intake of fish, moderate amounts of poultry, and low amounts of red meat, together with olive oil as the main source of fat. It is also characterized by a moderate intake of alcohol (primarily in the form of red wine and generally during meals), together with regular physical activity. Such recommendations are commonly known in the form of the “novel MD pyramid” (*Fundación Dieta Mediterránea*, *Spain*) (Figure 1). Although typical “Western” and Mediterranean diets might display a similar total fat content, the MD components contain essential nutrients such as monounsaturated fat with health-promoting properties, while the Western diet is high in saturated and trans fats [9,10,11]. The acquisition of foreign foods, time-compression, and sedentary practice could have counteracting effects on the local habits and, thus, in adherence to the MD. In fact, developed nations such as Greece or Italy are slowly replacing their traditional patterns of eating with a Western diet rich in animal products, refined carbohydrates, high-fat, and less consumption of fruits and vegetables [12,13,14].

Several nutritional studies have focused on the traditional cultural model and lifestyle of the Mediterranean Basin, comparing food, nutrition, and physical activity patterns. Nonetheless, there is a surprising paucity of nationwide studies, considering the influence of modern-day life on individual dietary habits in the Mediterranean area in general and in particular geographical regions. For this reason, the clinical study “Adherence to the MD in Spain and its relationship with the cardiovascular risk” (DIMERICA study) quantifies eating patterns in five geographic regions of Spain in order to check their real adherence to the MD and any association with potential cardiovascular risks. The findings should serve as a valuable source of information from a public health perspective, enabling a more descriptive analysis of the different eating habits in the Spanish population.

## 2. Methods

### 2.1. Study Design and Population

**Figure 2:**
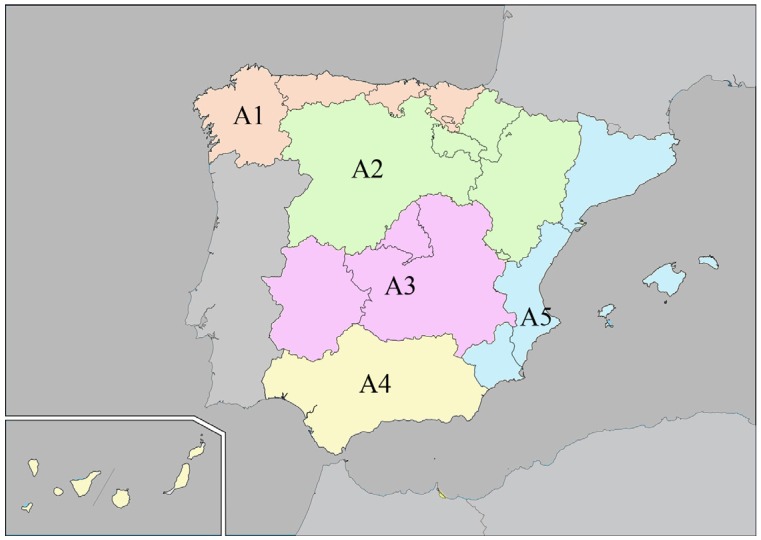
Map of Spain with the five studied areas. Area 1 (A1, orange); Area 2 (A2, green); Area 3 (A3, purple); Area 4 (A4, yellow); and Area 5 (A5, blue).

This contribution represents a descriptive cross-sectional study of national scope. Volunteers willing to participate were recruited from across Spain during spring/summer of 2012 and organized into five different areas according to geographical region (Figure 2). Area 1 (A1) includes the northern regions: *Galicia*, *Asturias*, *Cantabria*, and the Basque Country, all on the Atlantic coast, and the *Cordillera Cantábrica*. Area 2 (A2) covers the central regions: *Castilla y León*, *Navarra*, *Rioja*, and *Aragón* (central–north). Area 3 (A3) represents the heart of the Peninsular Spain: *Madrid*, *Castilla La Mancha*, and *Extremadura*. Area 4 (A4) is mainly composed of the Andalusian region in the south of Spain, with a long coastal area, the Canary Islands. Finally, Area 5 (A5) is located in eastern Spain and bordered by the Mediterranean Sea: *Cataluña*, *Comunidad Valenciana*, *Murcia*, and the Balearic Islands.

**Figure 3:**
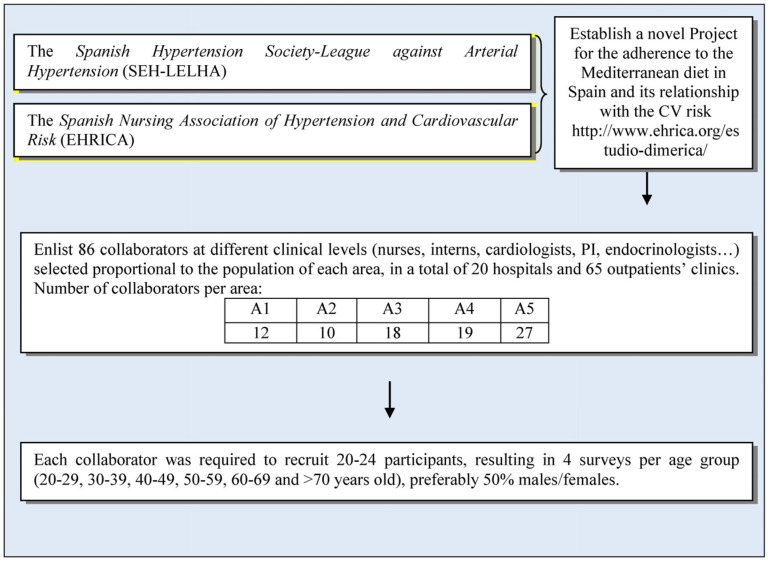
Process of volunteer recruitment.

The study was designed taking into consideration the census information of the National Institute of Statistics (*Instituto Nacional de Estadística*) for all the five areas. In 2010, Spain had 46,661,950 inhabitants, of whom 38,137,661 were >20 years old. The *Sociedad Española de Hipertensión-Liga Española para la Lucha contra la Hipertensión Arterial* (SEH-LELHA) (Spanish Hypertension Society-League against Arterial Hypertension) together with the *Asociación Española de Enfermería de Hipertensión y Riesgo Cardiovascular* (EHRICA) (Spanish Nursing Association of Hypertension and Cardiovascular Risk) provided 86 research collaborators covering all the regions, shown in Figure 2, taking into account the proportion of inhabitants of each area in relation to the total of the Spanish population. Collaborators had to recruit four participants for each established age group (20–29, 30–39, 40–49, 50–59, 60–69, and >70 years old) following the stratified pyramid of ages. In brief, the sampling scheme was a random three-stage stratified sampling exercise according to geographic area, age, and gender (Figure 3). Volunteers were recruited when they accompanied a relative to the different health centers where the collaborators worked. We used a randomized criterion during the selection of the participants, and less than 20% of the initially selected individuals declined to participate in the study. Sample size determination was calculated to achieve a confidence interval of 95%, alpha level of 0.05, and a desired level of precision of 2.5%, which gave a total sample size of 1537. The study, however, managed to recruit 1732 participants considering the proportions of inhabitants of each geographical area. A total of 86 collaborators were involved in the study: 12 from A1, 10 from A2, 18 from A3, 19 from A4, and 27 from A5. The research team met three times in order to unify the recruitment criteria and training, to ensure lack of bias in selection and methodological quality (Figure 3).

The study protocol and survey instrument were approved by the 12 de Octubre Hospital (Madrid) Review Board for the protection of human subjects. Informed consent was obtained from all individual participants included in the study.

### 2.2. Survey and MD Score

All participants were asked to complete a survey designed to assess their exercise, dietary patterns and preferences. The present survey was adapted from the validated ATTICA study survey [13]. Since the questionnaire was designed for a Mediterranean population and the issues requested meet the criteria for clarity, simplicity, and neutrality, divided into subject areas and avoiding double issues, and consisting of closed questions (with mutually exclusive answers), validation in the Spanish population was not considered necessary, accepting the bias that this decision could produce [15]. We considered more important the early involvement of volunteers in the study using a validated questionnaire in a similar culture and with similar eating habits. In fact, there were no problems regarding the understanding of the questions for any of the participants, so quite possibly the answers would reflect real food consumption and life habits.

The survey had 40 questions and was divided into three sections. The first section (11 questions) asked for anthropometric and demographic data, and reported cardiovascular risk factors (smoking status, physical activity, sedentary lifestyle) and level of education. The second section (14 questions) asked participants about their current dietary habits and preferences. The third section (15 questions) asked participants about their MD adherence. Estimated portion sizes were shown to the participants using colored pictures. All the collected data (dietary habits, clinical risk factors, etc.) were declarative. The survey took approximately 10 min to complete and was assessed by the collaborators in primary care centers and hospitals. To avoid reporting bias, volunteers were informed that the medical history could be consulted at the time of the survey.

To create a tailored MD adherence score, we examined data from the third section, and scored with one point each of the questions below:

Question 1. Consumption of cereals. ≥5 portions per day.
Question 2. Consumption of fruits. ≥3 portions per day.
Question 3. Consumption of legumes. ≥2 portions per week.
Question 4. Consumption of vegetables. ≥2 portions per day.
Question 5. Consumption of fish. ≥3 portions per week.
Question 6. Consumption of poultry meat. 1 ≤ portions per week ≤ 4.
Question 7. Consumption of red meat. 1 ≤ portions per week ≤ 4.
Question 8. Consumption of dairy products. 3–4 portions per day.
Question 9. Consumption of different oils. Olive oil as preferred oil.
Question 10. Use of olive oil for cooking. Daily use.
Question 11. Consumption of nuts. >1 portion per week.
Question 12. Consumption of factory-baked products. <1 per week.
Question 13. Consumption of eggs. < 1 egg per week < 7.
Question 14. Consumption of cold-meat. <2 per week.
Question 15. Consumption of red wine. >1 glass per week.

The total score was multiplied by 10 and divided by 15 to obtain a value out of 10. This value represented the MD adherence as follows:

Score <5: Poor MD adherence, requiring improvement in dietary habits.
Score 5–7: Average MD adherence, which could be improved with small modifications.
Score >7: Good MD adherence.

Because the MD adherence score requires minimal information from the volunteer and no sensitive health information, it can be generated for members of any volunteer population. The general level of MD adherence received a favorable opinion by our Nutrition Department at the Catholic University of Murcia (UCAM).

### 2.3. Statistical Analysis

Data are expressed as mean (±standard deviation) for normally distributed data or median (25th percentile, 75th percentile) for non-normally distributed data. Frequencies were used as descriptors of the studied population. The Kruskal–Wallis or Friedman tests were used for non-normally distributed data to examine differences in frequencies of responses to questions on dietary and exercise habits. The *Z*-test with Bonferroni adjustment was used to compare percentages. Correlations between study parameters were assessed using Spearman’s method (for non-normally distributed parameters). A *p*-value of <0.05 was considered statistically significant. SPSS 18.0 software was used for statistical analyses (SPSS, Inc., Chicago, IL, USA).

## 3. Results

### 3.1. Baseline Characteristics of the Cohort and Lifestyle

The baseline characteristics of the participants divided by area are shown in Table 1. A total of 1732 subjects were included in the analysis (47% males; 51 (37–65) years old). Median (interquartile range) anthropometric values were: weight 72 (62–82) kg and body mass index (BMI) 25.6 (22.7–29.2) kg/m^2^. Thirty five percent of the population had a BMI of between 25 and 29.9 kg/m^2^ based on self-reported height and weight data, indicating a high percentage of overweight individuals. Moreover, 21% of individuals in the population were defined as obese (BMI ≥ 30).

When baseline characteristics were compared according to the five geographical areas, no significant differences were found concerning age, sex, weight, height, body mass index, waist circumference, and trouser size (Table 1). However, differences were evident for obesity, smoking, and hypertension (*p* = 0.04, *p* = 0.005, and *p* = 0.012, respectively). Pairwise comparisons found that A4 displayed a lower prevalence of hypertension than A1 (*p* = 0.016) and A3 (*p* = 0.017). Similarly, A2 presented a higher proportion of smokers than A3 and A4 (*p* = 0.013 and *p* = 0.008, respectively). No differences were found concerning diabetes and dyslipidemia prevalence among the different areas.

Participants were asked how often they practice exercise, including going up and down steps. Thirty nine percent reported practicing exercise daily, while 23.1% did not (Table 2), and no gender differences were found (data not shown). The survey also showed that 22.9% of the cohort watched television for less than 30 min a day, 42.6% between 1 and 2 h daily, and 15.3% more than 2 h daily (Table 2). Habits were common to all the areas and genders (data not significant).

### 3.2. Mediterranean Diet in the Total Population

Participants were asked about their consumption of foods, frequency of servings, and alcohol intake. Cereals and their derivates (bread, rice, and starchy food) were widely consumed among the Spanish population. Forty percent ate 3–4 cereal portions on a daily basis and 44.6% between 1 and 2 portions (Table 3). Nevertheless, such servings were relatively insufficient according to the MD recommendations (>7 servings).

The survey found that half of the population consumed 1–2 pieces of fruit per day, whereas only 30% followed the MD recommendations (3–4 fruits). Consumption of fruits, vegetables, and legumes was high among the population but still insufficient; it was higher among women (all *p* < 0.005, data not shown). Dairy products were also very common in the diet and 64% reported to consume them daily; again, women ate more dairy products than men (*p* < 0.001, data not shown).

Another important issue was fish intake, and this was consumed less than recommended across all five areas. Only 30.2% of the population ate 3–4 portions of fish per week. Similarly, poultry intake was also lower than recommended—once or twice per week in half of the general population (54.7%), and only 31.6% reported consumption of 3–4 portions per week, as recommended. Interestingly, red meat was eaten as often as white meat since 55.9% also had it once or twice weekly. Eggs were very often consumed and 57.4% had them once or twice per week (data not shown). Moderate red wine intake was not widely consumed, as half of the population reported never to drink red wine (48.4%) and only 9.0% followed the MD recommendations of 4–7 glasses weekly (Table 3). It is important to note that 93.2% of the population consumed olive oil daily and 89.9% used olive oil for cooking. Eating nuts was not common, as 38.8% reported eating less than once per week and only 10% consumed them regularly.

Factory-baked products were not normally consumed by the population. By contrast, consumption of cold meat (processed sausages, etc.) was high, with 34.2% reporting consumption more than 3 times weekly and only 19.5% less than once per week, as recommended (Table 3).

Regarding the consumption of alcohol, other than red wine, 30% of individuals consumed alcoholic beverages less than once per week and 15.9% had 1–2 drinks per week. Men consumed higher quantities of alcohol than women (*p* < 0.001) (data not shown). Beer was one of the most consumed alcoholic beverages, with 22.5% of the population consuming 1–2 drinks per week and 19.1% more than 3 times per week. Most of the population tended to have 1–2 coffees per day (44.5%), 13.8% had more than 3 per day, and 22.6% never drank coffee (data not shown).

### 3.3. Mediterranean Diet in the Different Areas Studied

To determine whether differences in MD adherence exist among the general Spanish population, we compared the dietary habits and servings across areas (Table 4). Data showed that A1 has a greater daily consumption of cereals than other areas (all *p* < 0.01) and A5 consumed less fruits than the rest of Spain (all *p* < 0.05). Legumes were not often consumed in A3 or A5, where only 16.1% and 16.6%, respectively, consumed 3–4 servings per week, which was lower than the rest of the country (both *p* < 0.001). By contrast, red meat was regularly consumed. Although red meat was lower in A4 than in A1, A2, and A3 (all *p* < 0.02), 64.5% of the population consumed red meat at least once per week. A1 and A2 (northern regions) consumed more than 3 eggs per week on average (37%), which was higher than the rest of the population (*p* < 0.001). No differences were observed concerning poultry and dairy products intake among the different areas (*p* = 0.39 and *p* = 0.062, respectively). Fish intake was significantly different among the Spanish population (*p* < 0.001) and was lower in A5 than in A3 (*p* < 0.001).

### 3.4. Dietary Preferences

We also asked questions on dietary preferences. The analysis found that 44.8% of the population liked oily fish, 46.7% liked white fish, and 8.4% disliked fish. Interestingly, 16.5% of the cohort was on a diet at the moment of the survey. Related to the term “functional food”, indicating food that provides health benefits (e.g., prebiotics, calcium/sterols-enriched dairy products, etc.), only 13.2% of the total cohort recognized eating such products regularly, suggesting that they had sufficient knowledge of the term, while 42% knew nothing about functional food. This was particularly true in the region A5, where 79% of participants were unaware of the nature of “functional food”, which was significantly lower than for the rest of the cohort (all *p* < 0.01). The study also found that in general, the Spanish population does not clearly distinguish between skimmed and non-skimmed dairy products since only 32.23% bought only skimmed dairy products and 20.2% occasionally.

### 3.5. MD Adherence and Cardiovascular Risk Factors Prevalence

When adherence to the MD was evaluated, the overall score was 4.6 (3.3–6.0) points out of 10 (0 points was considered noncompliance, 10 points maximum compliance). Area 5 had the worst score, which was significantly lower than that achieved by A1, A2, and A4 (all *p* < 0.001) (Table 5). The center of Spain (A3) also presented low compliance compared with the rest of Spain. No differences were evident between genders in this respect (data not shown).

Arterial hypertension was associated with other risk factors such as diabetes, dyslipidemia, being overweight, and smoking (all *p* < 0.05). Furthermore, data from the survey showed that hypertension was also associated with the MD adherence score, being lower among hypertensive individuals (*p* = 0.017), whereas participants showing better MD adherence (score ≥ 7) had a lower prevalence of hypertension (*p* = 0.009) (Table 6). In the population as a whole, the lower consumption of fruits, poultry, and fish was also related with hypertension, as shown in Table 6 (all *p* < 0.05). However, after mutually adjusting for confounding variables, this association was no longer noted for a high adherence score, regular consumption of fruits and poultry (3–4/week). As shown in Table 7, increased fish (>3 times per week) and lower red meat (<3–4 times per week) intakes were still associated with a significantly decreased likelihood of hypertension (odds ratio (OR) 0.70, 95% confidence interval (CI) 0.52–0.97 and OR 0.68, 95% CI 0.49–0.96, respectively).

## 4. Discussion

Adherence to the MD is a multidimensional nutritional approach, as it involves not only dietary patterns but also lifestyle, sociocultural, cultural heritage, and environmental aspects [16]. Moreover, food dietary patterns are a major environmental factor and people are exposed to them numerous times every day during their lifetimes.

The study “Seguimiento de la Dieta Mediterránea y su relación con el Riesgo Cardiovascular en España (DIMERICA)” promoted by the *Sociedad Española de Hipertensión-Liga Española para la Lucha contra la Hipertensión Arterial* (SEH-LELHA) and EHRICA (*Enfermería de Hipertensión y Riesgo Cardiovascular*) (see Methods) aimed to monitor compliance with the MD in different geographic regions of Spain in order to identify “hot spots” for cardiovascular prevention. A total of 1732 adult participants from five different areas of Spain—north (A1), lower north (A2), center (A3), south (A4), and southeast (A5)—answered a questionnaire on how closely they followed the MD.

### 4.1. Definition of MD

The different definitions of the MD could limit our understanding of the mechanisms by which the MD confers its health benefits. One reason for this may be differences in the food servings or the use of different adherence scores by different studies. According to a recently published comparison, our MD pattern closely resembles to the recommendations of the *Mediterranean Diet Foundation* (2011), with flexible servings without specific amounts (e.g., grams of bread) [17,18].

### 4.2. Dietary Patterns per Area

The present study shows that, in general, the Spanish population displays poor nutritional habits that do not fit the MD recommendations, which is in line with previous reports [19,20]. Further, individuals from most of the areas failed to fulfill the recommendations concerning the minimum consumption of fruits, legumes, and vegetables, so the intake of carbohydrates was far from the MD pattern. For example, despite living in one of the leading areas for the export of fruits and horticultural products in Europe, the inhabitants of A5 had the lowest intake of fruits among the whole population. Conversely, A1 and A2 (north and lower north, respectively) had a higher intake of whole-grain cereals, but this was still insufficient by MD standards (40%, 3–4 times per day). Therefore, as a general rule, it seems that the consumption of plant foods should increase across Spain, but particularly in the Eastern regions. These data contrast with reports suggesting that the MD was closely followed in Spain and specific areas of the country [21,22,23].

Our data showed that proteins and fats in the form of animal products replaced the carbohydrate proportion of the diet. No differences were found concerning the intake of white meat and dairy products for the different regions. However, 47% of A2 participants (lower north) consumed red meat more than 3 times per week, which approximately doubles MD guidelines and could lead to the harmful intake of saturated fat. Although an analysis of the proportion of each macronutrient in the diet (carbohydrates, proteins and fats) was beyond the scope of the present study, a higher total fat content was evident (>35%) among the Spanish population. Studies in other Mediterranean countries have also documented such increase in the intake of total fat [13]. One favorable finding of the current survey is that increasing “westernization” of the diet does not seem to affect the use of olive oil as the main fat in the diet, since 90% recognized using it daily. However, it is uncertain to what extent virgin or extra-virgin olive was used. The latter oil could contribute further beneficial phenolic antioxidant effects. Together with olive oil, fish represents an important source of unsaturated fats. Unfortunately, fish consumption was below recommended guidelines. Indeed, it was noticeable and intriguing how A5 (Mediterranean area) presented the lowest fish intake. Finally, the results of the present study showed that red wine consumption is not widespread in the Spanish population as a whole; however beer and spirits were commonly consumed. All these results contributed to the low adherence score of the entire sample, which barely reached a compliance score of 5 out of 10. Area 5 presented the lowest MD adherence score because of its low intake of fish, plant products, and nuts.

### 4.3. Hypertension and Relationship with MD

There is a wealth of evidence that supports the inverse association between following the MD and the prevalence of coronary heart disease and hypertension [24,25,26,27]. It is important to note that the present study did not consider clinical data but only information directly declared by the volunteers. Despite this, a negative association was found between hypertension prevalence and fulfillment of the MD, in agreement with recent reports [25,28]. However, no relationship was reported between other risk factors (obesity, diabetes, dyslipidemia, and smoking) and MD compliance. Other European studies have recently assessed the adherence to the MD. Among them, the ATTICA retrospective study determined that MD inversely correlated with a higher risk of CVD events [13]. A Turkish study also related CV events with lower MD adherence in men [29]. However, each report used a different score for MD adherence—MedDietScore and score-MD, respectively. Our overall analysis, which includes all individuals, suggests that MD is closely related with cardiovascular risk factors such as hypertension. In fact, the MD pattern has been proposed as one of the best models for healthy eating because of its favorable inverse relationship with cardiovascular risk factors and outcomes [30]. Accordingly, the diet is frequently used to treat multifactorial diseases and traditional cardiovascular risk factors [31].

### 4.4. Cardiovascular Risk Factors and MD Score

The distribution of cardiovascular mortality is uneven in Spain, being higher among the Mediterranean regions and islands when compared with north–central Spain [32]. Despite the strength of the evidence, the clinical explanation for the same is still unknown. Generally, it would be thought that Mediterranean regions would dutifully follow a MD pattern, and would have better cardiovascular outcomes than the rest of Spain. However, the MD adherence scores found do not reflect this notion, since they were lower in the Mediterranean area. Therefore, it is plausible and indeed likely that the differences in mortality observed between regions are related with a lower compliance with the MD, although other factors must also intervene. This underlines the idea that better data about the intake of major food groups in Spain are needed to gain a more accurate picture of how changes in consumption affect cardiovascular risk and to guide future health policy [33].

### 4.5. Limitations of the Study

Our study has limitations that warrant discussion. We did not validate the questionnaire, although it was previously validated in another Mediterranean population. This was not done based on the clarity, simplicity, and other positive properties of the initial survey, as indicated in Methods. Additionally, the study lacks clinical, biochemical, and socioeconomical data which would complement the self-reported—and possibly subjective—information provided. However, other studies with self-reported information have found associations between adherence to the MD and cardiovascular risk factors [34]. The diagnosis of CVD risk factors does not represent a bias simply because the medical history could have been consulted at the time of the survey. Nonetheless, potential biases are in the same direction (under or overestimation) so that the relationships between variables can be admitted and the conclusions considered acceptable.

The fact that the participants were randomly selected in healthcare centers when accompanying their relatives should be born in mind when extrapolating the results to the general population.

## 5. Conclusions

The present study gives an account of the poor level of MD adherence in the general population and specific areas of Spain. It seems that while other countries in the world are seeking to adopt the MD, Spain, paradoxically, is abandoning it. The poor MD adherence observed is in line with the high prevalence of hypertension observed and, therefore, with increased cardiovascular risk. Our study also highlights the importance of olive oil consumption, which sustains higher MD adherence among the whole country. It is clearly essential to provide corrective measures and health guidelines in relation to food and nutrition, even in a country famed for its classically Mediterranean diet. A better understanding of the benefits of dietary habits is necessary to address the problems of cardiovascular diseases.

## Figures and Tables

**Table 1:** Demographic/anthropometric characteristics of the total population organized by geographical areas (total population *n* = 1732).

	Total Population (*n* = 1732)	Area 1 (*n* = 221)	Area 2 (*n* = 149)	Area 3 (*n* = 621)	Area 4 (*n* = 277)	Area 5 (*n* = 464)	*p* Value
Age	51 (37–65)	50.0 (36–65)	49.0 (36–64)	52.0 (38–66)	50.0 (36–64)	51.0 (37–65)	0.56
Males, %	47%	50.0%	47.4%	44.8%	49.5.5%	46.8%	0.77
Weight (kg)	72 (62–82)	73 (64–84)	70 (59–80)	72 (62–82)	72 (62–84)	71 (61–81.5)	0.06
Height (cm)	166 (160–173)	166 (161–173)	167 (160–174)	165 (159–172)	168 (160–174)	165 (160–173)	0.07
Body mass index category (kg/m^2^)	25.6 (22.7–29.2)	25.7 (22.8–29.8)	24.8 (21.9–28.1)	25.8 (22.8–29.7)	25.6 (22.8–29.2)	25.4 (22.3–28.5)	0.09
Normal weight (18.5–24.9 kg/m^2^)	43.8%	43.8%	48%	46%	42.7%	45.9%	0.31
Overweight (25.0–29.9 kg/m^2^)	35.2%	31.6%	33.5%	35.5%	36.7%	34.9%	0.31
Obese (BMI ≥ 30.0 kg/m^2^)	21%	24.6%	18.5%	14.5%	20.6%	19.2%	0.04
Waist circumference (cm)	92 (82–101)	91 (82–103)	90 (79–99)	93 (81–103)	93 (83,–102)	90 (83–99)	0.11
Trouser size	44 (40–48)	44 (40–48)	42 (40–46)	44 (40–48)	44 (40–48)	43 (40–48)	0.07
Smoking, %	19.2%	17.9%	21.7%	18.2%	18.5%	20.7%	0.005
Dyslipidemia, %	21.5%	20.1%	16.4%	22.3%	21.7%	22.5%	0.54
Diabetes mellitus, %	10.4%	9.8%	8.6%	10.1%	12.1%	9.8%	0.95
Hypertension, %	25.5%	30.4%	23.0%	27.7%	17.8%	25.5%	0.012
University Degree, %	44.2%	39.3%	42.8%	42.9%	48%	45.5%	0.48

Data are presented as median (interquartile range) for non-normally distributed variables. Comparisons are assessed by Kruskal-Wallis test.

**Table 2:** Lifestyle habits in the total population (*n* = 1732).

	Total (*n* = 1732)	Area 1 (*n* = 210)	Area 2 (*n* = 147)	Area 3 (*n* = 617)	Area 4 (*n* = 262)	Area 5 (*n* = 456)	*p* Value
**Physical activity level, %**							
Daily, %	39.5	37.5	34.9	35.8	33.5	32.4	NS
4–5 times per week, %	12.9	10.3	9.9	12.9	8.9	11.7	NS
2–3 times per week, %	24.1	20.1	16.4	20.8	20.3	24.0	NS
Never (sedentary), %	23.1	18.8	26.3	16.6	24.2	21.7	NS
**Watching Television, h/day, %**							
<1, %	22.9	19.2	23.1	20.6	19.6	25.0	NS
1–2, %	42.6	42.9	45.4	37.5	38.4	40.7	NS
≥2, %	34.2	31.7	28.5	33.4	35.2	29.4	NS

NS: nonsignificant.

**Table 3:** Mediterranean intake habits in the total population (*n* = 1732).

	**>7**	**4–6**	**3–4**	**1–2**	**None**
Cereals per day, %	3.6	10.7	39.9	44.6	1.1
Fruits per day, %	2.0	7.2	29.9	53.9	6.9
	**>2**	**2**	**1**	**<1**	**none**
Vegetables per day, %	9.5	29.8	45.2	14.2	1.2
	**>4**	**3–4**	**1–2**	**<1**	**none**
Legumes weekly, %	2.0	20.1	62.0	12.8	2.4
Fish weekly, %	5.5	30.2	54.0	8.7	1.7
White meat weekly, %	5.6	31.6	54.7	6.0	2.1
Other meat weekly, %	4.4	18.7	55.9	15.8	5.2
Dairy products daily, %	4.5	28.5	63.9	1.9	1.0
	**>7**	**4–7**	**1–3**	**<1**	**none**
Red wine (in moderation), weekly,%	5.8	9.0	13.6	22.9	48.4
	**Daily**	**3–5**	**1–2**	**<1**	**none**
Olive oil for cooking weekly, %	89.9	4.4	2.6	1.5	1.6
Factory-baked products weekly, %	5.2	7.1	17.7	34.7	35.2
	**>5**	**3–4**	**1–2**	**<1**	**none**
Nuts weekly, %	6.2	10.0	25.9	38.8	19.1
Cold-meat weekly, %	9.5	24.7	36.1	19.5	10.2

**Table 4:** Regional difference of the consumption of key components of Mediterranean Diet.

	Area 1 (*n* = 221)	Area 2 (*n* = 149)	Area 3 (*n* = 621)	Area 4 (*n* = 227)	Area 5 (*n* = 464)	*p* Value
Cereals, >5/day, %	21.9	15.1	13.4	14.5	13.1	<0.001
Fruits, >3/day, %	40.4	36.1	42.6	40.4	30.5	<0.05
Legumes, 3–4/week, %	33.2	23.2	16.1	24.5	16.6	<0.001
Red meat, <1/week, %	22.6	13.4	20.7	35.5	26.2	<0.001
Poultry, 3–4/week, %	29.8	27.7	28.9	34.1	35.9	0.39
Eggs, >3 week, %	49.0	48.9	29.6	40.5	27.9	<0.001
Fish, >3 week, %	35.6	35.5	41.2	36.9	27.2	<0.001
Dairy products, daily, %	61.9	66.0	63.4	59.5	68.1	0.062

*Z* test for proportions with Bonferroni adjustment.

**Table 5:** MD adherence according to score (out of 10) in the different areas of Spain.

	Area 1 (*n* = 221)	Area 2 (*n* = 149)	Area 3 (*n* = 621)	Area 4 (*n* = 277)	Area 5 (*n* = 464)	*p* Value
Score	5.3 (4.0, 6.0) *^,†^	5.3 (4.0, 6.0) *^,†^	4.6 (3.3, 6.0)	5.3 (4.0, 6.0) *^,†^	4.6 (3.3, 4.6)	<0.001

Comparisons between 5 Areas were assessed by Kruskal-Wallis test. * Mann–Whitney test with Area 3, *p* < 0.005. ^†^ Mann–Whitney test with Area 5, *p* < 0.001.

**Table 6:** Prevalence of hypertension according to other cardiovascular risk factors and main dietary patterns in the entire population.

	Hypertension (*n* = 462)	No Hypertension (*n* = 1239)	*p* Value
Diabetes mellitus, %	22.8	6.3	<0.05
Dyslipidemia, %	44.1	19.4	<0.05
Smoking, %	19.2	28.3	<0.05
Overweight, %	81.6	44.6	<0.05
Adherence score	4.6 (3.3–6.0)	5.3 (4.0–6.0)	0.017 ^†^
Adherence score ≥ 7, %	7.9	11.5	<0.05
Poultry, 3–4/week, %	30.3	35.9	<0.05
Red meat, 3–4/week, %	27.2	19.2	<0.05
Fish, >3 week, %	28.5	35.5	<0.05
Fruits, 3–4/daily, %	27.9	34.3	<0.05

**^†^** Mann–Whitney test. *Z* test for proportions with Bonferroni adjustment.

**Table 7:** Association between hypertension and dietary intake.

Covariate	Unadjusted OR (95% CI)	Adjusted OR ^a^ (95% CI)	Adjusted OR ^b^ (95% CI)
Adherence score ≥ 7	0.66 (0.47–0.94) *	1.02 (0.63–1.61)	0.84 (0.58–1.13)
Fruits, 3–4/daily	1.03 (0.76–1.28)	0.84 (0.63–1.13)	
Poultry, 3–4/week	0.82 (0.66–1.02)	0.77 (0.57–1.03)	
Fish, >3 week	0.78 (0.63–0.98) *	0.71 (0.52–0.97) *	
Red meat, <3–4/week	0.68 (0.53–0.87) *	0.68 (0.49–0.96) *	

OR: odds ratio, CI: confidence interval; ^a^ Mutually adjusted OR (in addition for dyslipidemia, diabetes mellitus, smoking, and overweight (BMI > 25 kg/m^2^)); ^b^ Adjusted OR for dyslipidemia, diabetes mellitus, smoking, and overweight (BMI > 25 kg/m^2^); * *p* < 0.05.

## Abbreviations

A1	Area 1
A2	Area 2
A3	Area 3
A4	Area 4
A5	Area 5
CV	Cardiovascular
CVD	Cardiovascular Disease
DIMERICA	Dieta Mediterránea y su relación con el Riesgo Cardiovascular en España (Mediterranean Diet and its relation with Cardiovascular Risk in Spain)
EHRICA	Asociación Española de Enfermería de Hipertensión y Riesgo Cardiovascular (Spanish Nursing Association of Hypertension and Cardiovascular Risk)
MD	Mediterranean diet
SEH-LELHA	Sociedad Española de Hipertensión-Liga Española para la Lucha contra la Hipertensión Arterial (Spanish Hypertension Society-League against Arterial Hypertension)
